# Retraction Note to: Linc00152 promotes malignant progression of glioma stem cells by regulating miR-103a-3p/FEZF1/CDC25A pathway

**DOI:** 10.1186/s12943-022-01660-3

**Published:** 2022-09-29

**Authors:** Mingjun Yu, Yixue Xue, Jian Zheng, Xiaobai Liu, Hai Yu, Libo Liu, Zhen Li, Yunhui Liu

**Affiliations:** 1 Department of Neurosurgery, Shengjing Hospital of China Medical University, 110004 Shenyang, People’s Republic of China; 2 Liaoning Clinical Medical Research Center in Nervous System Disease, 110004 Shenyang, People’s Republic of China; 3 Key Laboratory of Neuro-oncology in Liaoning Province, 110004 Shenyang, People’s Republic of China; 4 Department of Neurobiology, College of Basic Medicine, China Medical University, 110122 Shenyang, People’s Republic of China; 5 Key Laboratory of Cell Biology, Ministry of Public Health of China, China Medical University, 110122 Shenyang, People’s Republic of China; 6 Key Laboratory of Medical Cell Biology, Ministry of Education of China, China Medical University, 110122 Shenyang, People’s Republic of China

**Retraction note to:** Mol Cancer **16**, 110 (2017)

10.1186/s12943-017-0677-9.

The Editor-in-Chief has retracted this article. After publication, concerns were raised regarding partial image overlap in Figs.1, 2 and 5–7. The Editor-in-Chief therefore no longer has confidence in the presented data.

Yunhui Liu has stated on behalf of all co-authors that they do not agree to this retraction.

